# The Mg doping ZIF-8 loaded with Icariin and its antibacterial and osteogenic performances

**DOI:** 10.1007/s10856-023-06755-x

**Published:** 2023-10-12

**Authors:** Lili Li, Jianghui Zhao, Fengcang Ma, Daihua He, Ping Liu, Wei Li, Ke Zhang, Xiaohong Chen, Lin Song

**Affiliations:** 1 School of Materials and Chemistry, University of Shanghai for Science and Technology, Shanghai, 200093 China; 2 Passive Device Test Center, China Medical Device Test Center, Shanghai, 201318 China

## Abstract

In this study, ICA@Mg-ZIF-8 was synthesized by Mg doping in ZIF-8 and loaded with icariin (ICA). The morphologies and phases were observed and analyzed by SEM, XRD, and the release behaviors of Mg, Zn ions and ICA were tested. Its antibacterial and mineralization performances were evaluated. The results showed that ICA@Mg-ZIF-8 has the same morphology and crystal structure as ZIF-8. ICA@Mg-ZIF-8 showed enhanced antibacterial activity against *Escherichia coli* and *Staphylococcus aureus*, and the antibacterial rate was increased to 87.7 % and 64.0 %, respectively. The results of in vitro mineralization showed that ICA@Mg-ZIF-8 presented better osteogenic performance promoting the uniform deposition of more calcium and phosphorus in simulated body fluids compared to ZIF-8.

**Graphical Abstract:**

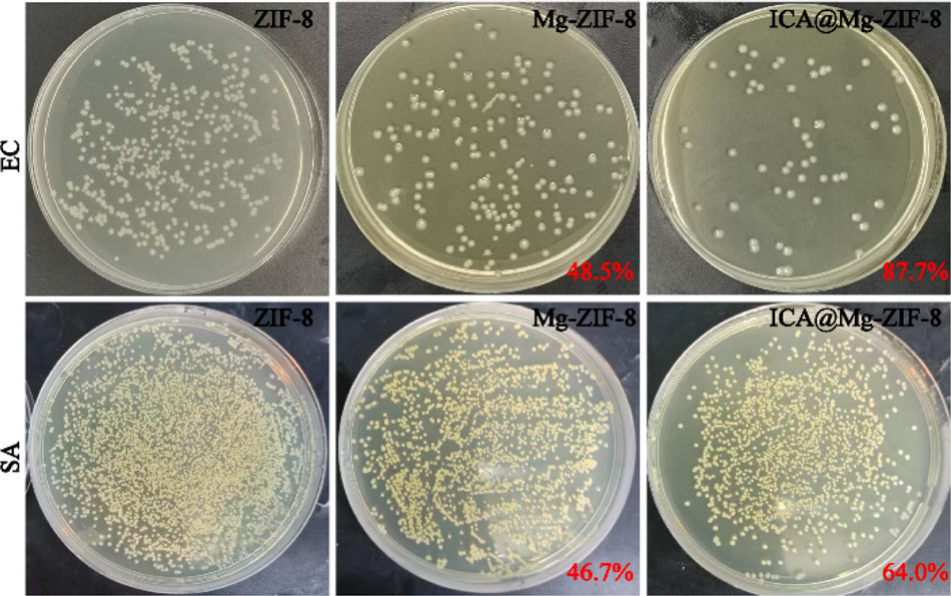

## Introduction

The lack of bioactivity and antibacterial capacity of the unmodified titanium implant surface may lead to a failure of implantation due to the loose of implant or bacterial infection of near tissue [1, 2]. Therefore, various bioactive and antibacterial coatings were usually prepared on titanium implant to improve its performances in clinical applications.

Zeolitic imidazolate framework-8 (ZIF-8) is a good biocompatible material with excellent thermal and chemical stability, and advantage for drugs delivering [3, 4]. Previous studies showed that ZIF-8 has antibacterial effect in acidic environment by releasing Zn^2+^ [5, 6]. The antibacterial mechanism of ZIF-8 is also through inducing alkaline microenvironment [7] and producing reactive oxygen species from the presence of metals [8]. Although ZIF-8 presents good antibacterial performance, its osteogenic bioactivity is not good enough for clinical application [9, 10]. How to improve the osteogenic bioactivity of ZIF-8 is still a problem to be solved for its application in titanium implant surface modification. With the invention of icariin (ICA), many researchers paid attention to its anti-inflammatory and osteogenic effect. Studies reported that ICA promoted periodontal tissue regeneration and has anti-inflammatory and immunomodulatory effects [11]. More evidences suggest that ICA can accelerate the repair of damaged bone tissue [12, 13]. Achieving long-term release of ICA after implantation is very helpful for bone repair to prevent the loose of implant. Kang et.al reported that magnesium organic frame-based scaffolds presented super antibacterial performance and promoted osteogenic differentiation activity [14]. Therefore, ZIF-8 doped with Mg^2+^ could be used to improve its antibacterial and osteogenic properties.

In this study, Mg^2+^ doping ZIF-8, then ICA was loaded to synthesize ICA@Mg-ZIF-8 nanoparticles. The synthesized ICA@Mg-ZIF-8 were characterized with SEM, XRD. The antibacterial activity of ICA@Mg-ZIF-8 against Escherichia coli and Staphylococcus aureus was tested, and the osteogenic activity was evaluated by in vitro mineralization.

## Experimental details

Stoichiometric 2-methyl imidazolae, zinc nitrate and magnesium nitrate were dissolved in deionized water, respectively. The 2-methyl imidazolae solution was poured into the magnesium nitrate solution and reacted for 5 min. Then the mixed solution was poured into zinc solution and stirred evenly. Finally, the ICA solution was poured into the mixture from the previous step and stirred. After 0.5 h, the final mixed solution was centrifuged at 8000 rmp for 40 min, and the centrifuged precipitation was dried at 80 °C for 10 h. Finally, ICA@Mg-ZIF-8 powder was obtained.

The surface morphology of the samples was characterized by SEM and the element distribution was analyzed by EDS. The phases and valence states of the samples were analyzed by XRD, XPS respectively. FT-IR spectra of the samples were recorded in the range 400–4000 cm^−1^. The releases of Mg^2+^ and Zn^2+^ were determined by ICP at 6, 12, 24, 48 and 96 h in the phosphate buffered saline. The pH value variation of the samples was measured in a beaker containing 10 mL normal saline at 37 °C after soaking for 6, 12, 18 and 24 h.

In vitro antibacterial experiments were performed with *Escherichia coli* (EC, ATCC25922) and *Staphylococcus aureus* (SA, ATCC29215). The antibacterial efficacy was calculated using the following equation:

$$ABE \% =\frac{{N}_{b}-{N}_{t}}{{N}_{b}}\times 100 \% .$$

Where *N*_b_, *N*_t_ was the number of viable bacterial colonies of ZIF-8, Mg-ZIF-8 or ICA@Mg-ZIF-8, respectively. In vitro mineralization experiment, the samples were immersed in simulated body fluids (SBF) for 7 days [15], then they were observed by SEM and element distribution on the surface were analyzed by EDS-Mapping.

## Results and discussions

### Morphology and phases of ICA@Mg-ZIF-8 nanoparticles

**Fig. 1:**
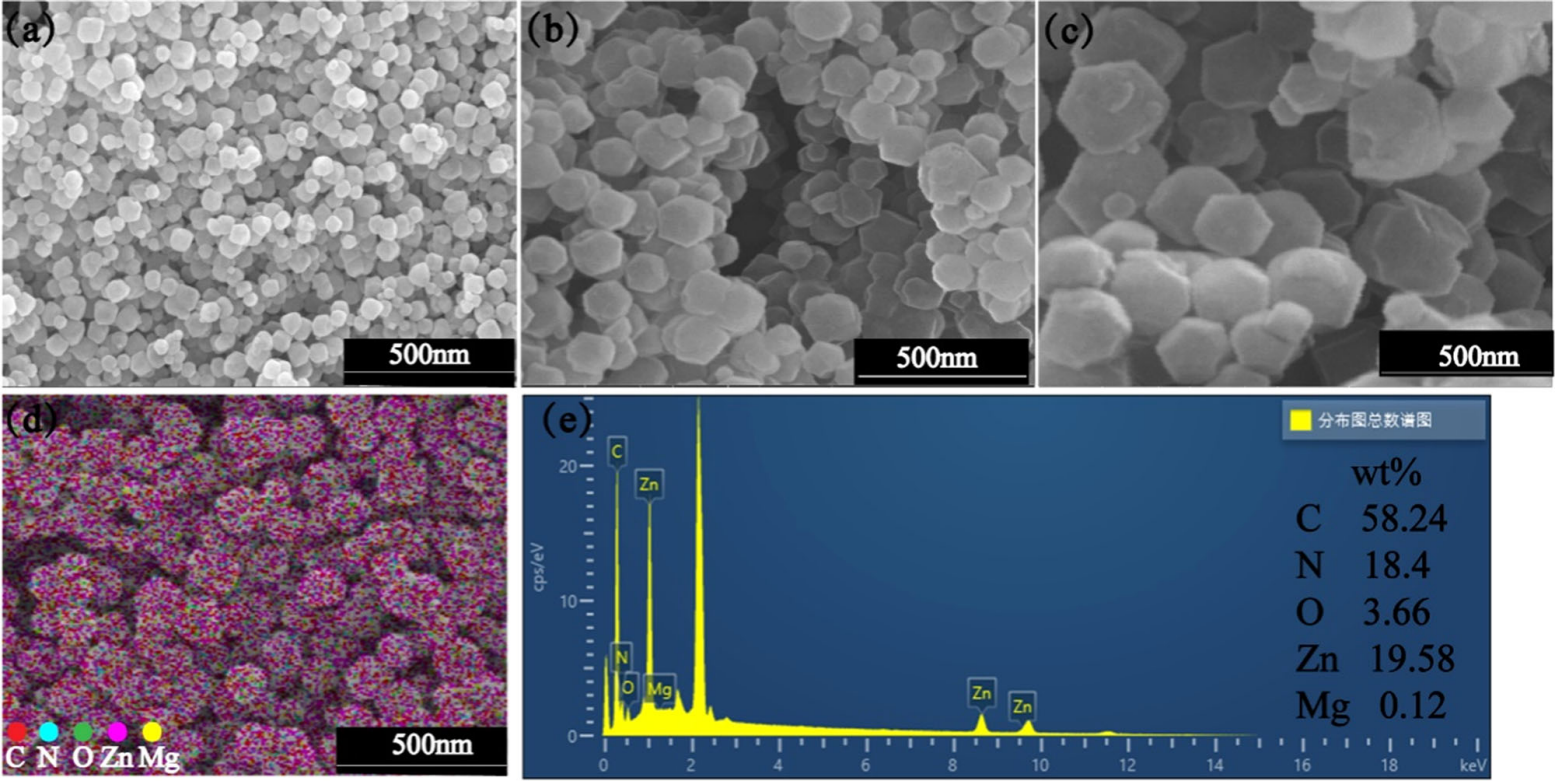
Morphologies of (**a**) ZIF-8, (**b**) Mg-ZIF-8 and (**c**) ICA@Mg-ZIF-8 nano-powders by SEM, (**d**) EDX-Mapping of the ICA@Mg-ZIF-8, (**e**) EDX spectrum of the ICA@Mg-ZIF-8

The morphologies of ZIF-8, Mg-ZIF-8 and ICA@Mg-ZIF-8 were observed by SEM. In Fig. 1a, ZIF-8 nanoparticles have a typical rhomboidal dodecahedron structure with particle size ranging from 200 to 400 nm. In Fig. 1b, c, the morphology of Mg-ZIF-8 and ICA@Mg-ZIF-8 was similar to that of ZIF-8. In this study, Mg^2+^ replaced some Zn^2+^ in lattice of ZIF-8. The synthesized Mg-ZIF-8 only introduced Mg^2+^ in ZIF-8 without the change of morphology, and the ICA@Mg-ZIF-8 loaded with ICA also had the same morphology only with the increase in particles size, the preservation of the complete ZIF-8 structure was observed. Generally, crystal growth includes nucleation and growth stages. According to Avrami’s crystal growth theory, for 3D crystals, the scale constant k can be expressed as *k* = (2*π*N_v_g^3^)/6, where *Nv* is the nucleation rate, *g* is the growth rate, and *k* is the scale constant [16]. The original ZIF-8 had a very fast nucleation rate, which resulted in the formation of small crystals [17]. During the growth of ZIF-8 crystals, the binding force of other metal ions with 2-methylimidazole ligands is weaker than that of Zn ions, resulting in a decrease in crystal nucleation rate [18]. The interaction of Zn ions with 2-methylimidazole ligands and the further growth of crystals are dominated by other metal ions coordinated by 2-methylimidazole ligands. In this study, the further growth of MOF particles confirms that Mg^2+^ and ICA coordinated with 2-methylimidazole participate in the further growth of crystals [17, 19, 20]. The Mg-ZIF-8 and ICA@MgZIF-8 nanocomposites were formed during the crystal growth process.

In Fig. 1d, e, The O element appears and was evenly distributed in ICA@Mg-ZIF-8. Because ICA is composed of three elements: C, H, and O, and Mg-ZIF-8 is composed of four elements: C, N, Zn, and Mg, it can be determined that ICA has been successfully loaded onto Mg-ZIF-8.

**Fig. 2:**
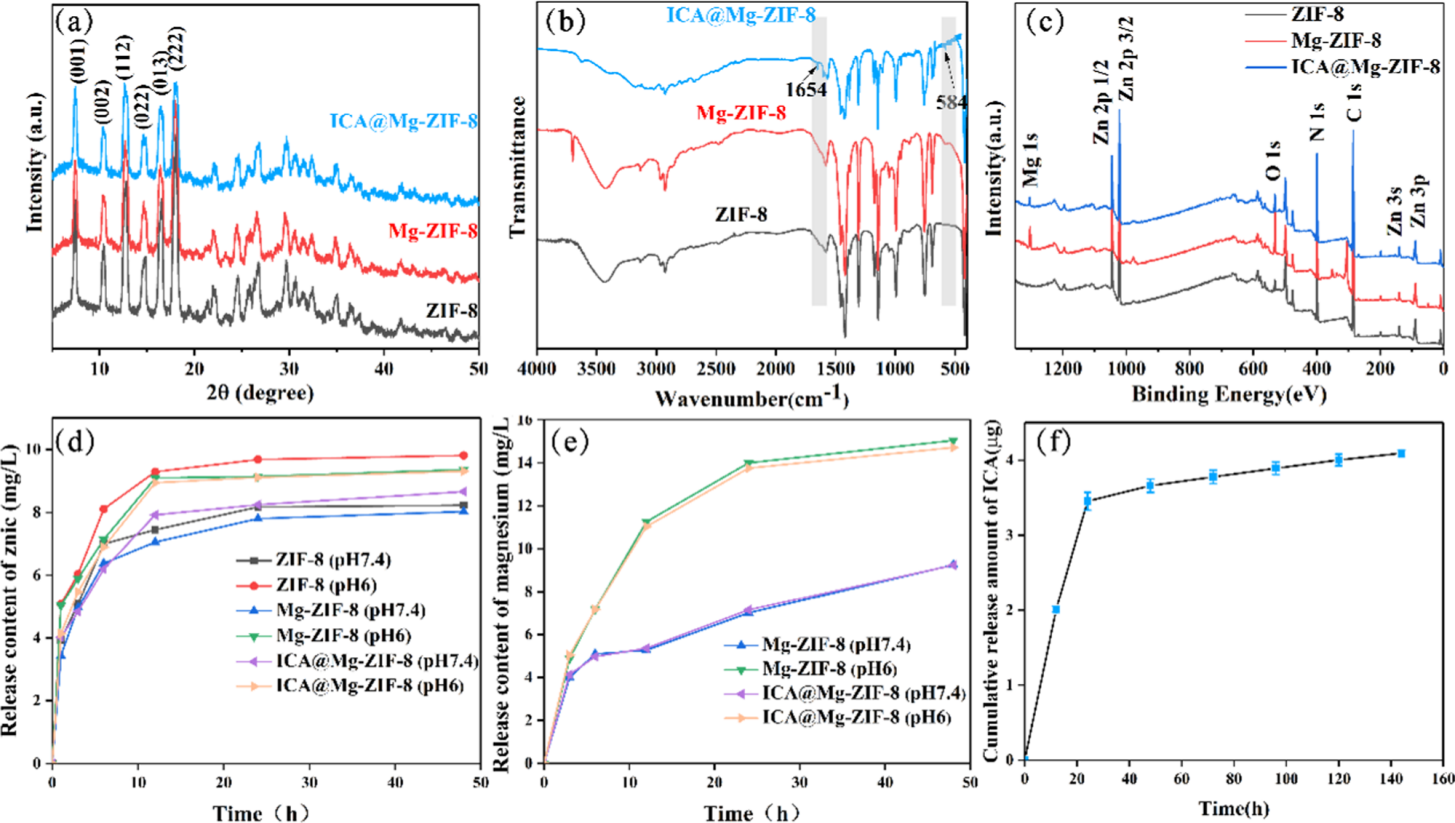
Characterization of ZIF-8, Mg-ZIF-8 and ICA@Mg-ZIF-8: (**a**) XRD patterns, (**b**) FT-IR spectra, (**c**) XPS full spectra, (**d**) Zn ions release, (**e**) Mg ions release, (**f**) ICA release of ICA@Mg-ZIF-8

XRD analysis results of ZIF-8, Mg-ZIF-8 and ICA@Mg-ZIF-8 are shown in Fig. 2a. The XRD patterns of Mg-ZIF-8 and ICA@Mg-ZIF-8 synthesized in this study correspond to the characteristic peaks (011), (002), (112), (022), (013) and (222) of the standard ZIF-8 showing ZIF-8, Mg-ZIF-8, and ICA@Mg-ZIF-8 with similar crystal structures. The reason is that Mg^2+^ replaced some Zn^2+^ in ZIF-8 crystal lattice but its crystal structure did not change, while ICA loading on Mg-ZIF-8, the crystal structure did not change either. The FT-IR spectra of ZIF8, Mg@ZIF-8 and ICA@Mg@ZIF-8 are shown in Fig. 2b. Mg@ZIF-8 and ICA@Mg@ZIF-8 shows characteristic peaks at 584 and 1654 cm^−1^, which are caused by the stretching vibration caused by Mg-N. Figure 2c shows the XPS survey spectra of ZIF-8, Mg ZIF-8, and ICA@Mg-ZIF-8 composites, in addition to the characteristic peaks of C, O, N, and Zn, there are also characteristic peaks of magnesium in Mg-ZIF-8 and ICA@Mg-ZIF-8 indicating that Mg was successfully doped into ZIF-8.

### Release of Zn, Mg ions and ICA of ICA@Mg-ZIF-8 nanoparticles

The release of Zn^2+^ and Mg^2+^ was detected for ZIF-8, Mg-ZIF-8 and ICA@Mg-ZIF-8, as shown in Fig. 2d, e. When pH is 7.4, Zn^2+^ and Mg^2+^ in nanoparticles were released slower compared to pH 6.0, which is attributed to a higher stability of ZIF-8 in alkaline or neutral environment than acidic condition [3, 4]. Free Zn^2+^ and Mg^2+^ can have positive effects on antibacterial and bone promoting properties, which will be reported in the next section. ICA release detection was conducted on ICA@Mg-ZIF-8, as shown in Fig. 2f. before 24 h ICA suddenly released and slowly released after that, this is because the ICA floating outside was released first, and then the ICA loading in Mg-ZIF-8 was release. The released ICA can promote the proliferation and differentiation of osteoblasts.

### Antibacterial and osteogenic performances of ICA@Mg-ZIF-8

**Fig. 3:**
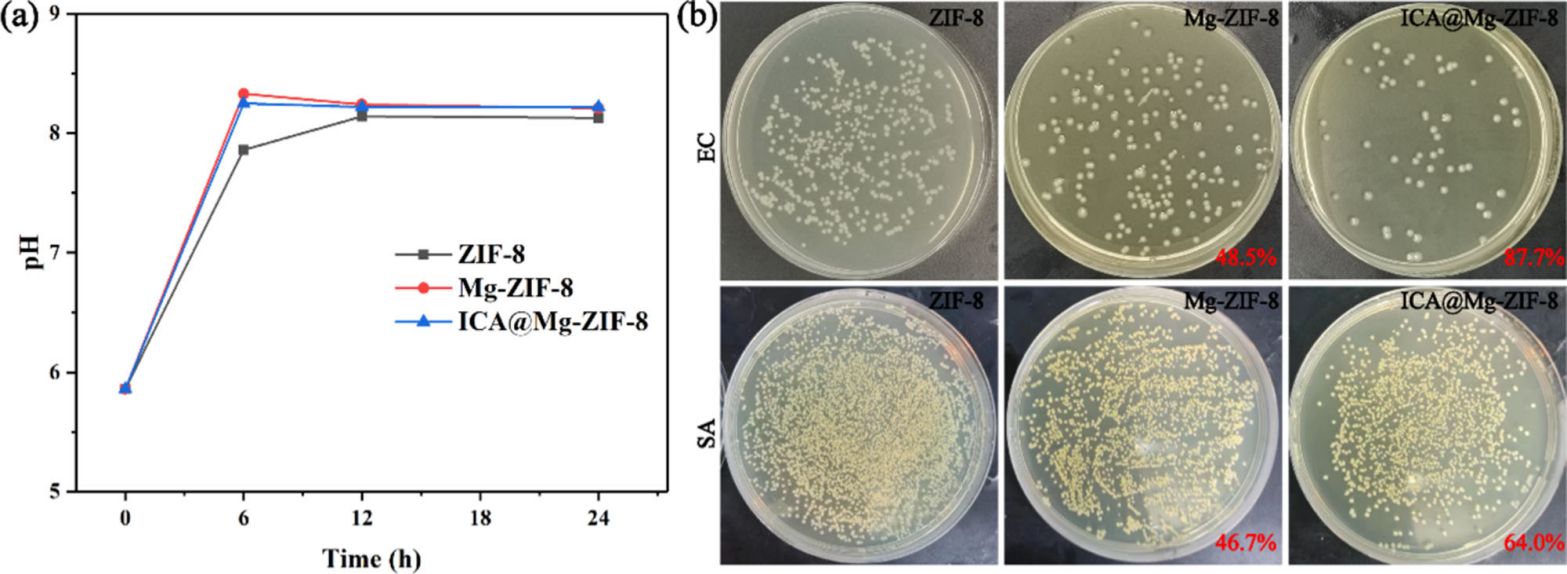
**a** pH value variation after various immersion time, (**b**) bacterial colony maps and inhibition rates of EC and SA on LB AGAR plates of ZIF-8, Mg-ZIF-8 and ICA@Mg-ZIF-8

Alkaline microenvironment can improve the activity of bone cell [21]. Meanwhile, the microenvironment with a pH value greater than 8.0 may lead to the death of bacteria [22]. Therefore, the pH value of prepared materials was examined, and the results were shown in Fig. 3a. The pH value of Mg-ZIF-8 and ICA@Mg-ZIF-8 was higher than that of ZIF-8, which may result from the doping of Mg ion. The CFUs of both EC and SA on LB agar culture plates after culturing for 24 h were shown in Fig. 3. Mg^2+^ doping can improve the antibacterial rates of both EC and SA, the antibacterial rate was 48.5% and 46.7%, respectively. The antibacterial rate of EC and SA increased after ICA loading, the antibacterial rate was 87.7 % and 64.0 %, respectively. Due to the synergistic effect of Mg^2+^ and ICA, ICA@Mg-ZIF-8 showed excellent antibacterial performance.

The primary antimicrobial activity of MOFs is due to the release of metal cations and organic ligands. Specifically, Zn^2+^ on ZIF-8 exhibit anti-inflammatory and antimicrobial properties [5, 6]. With the inclusion of Mg^2+^ and ICA, the diameter of ZIF-8 particles increases. ZIF-8’s high porosity and large surface area facilitate the loading of a significant amount of carrier and synergistic bacterial preparations into its pores, thereby enhancing its antimicrobial performance. Mg-ZIF-8 and ICA@Mg-ZIF-8 have a stronger inhibitory effect on EC than on SA, primarily due to differences in cell wall structure. Specifically, the cell wall of SA lacks lipids and proteins and exists in a slightly acidic environment, which promotes bacterial growth [12–14].

**Fig. 4:**
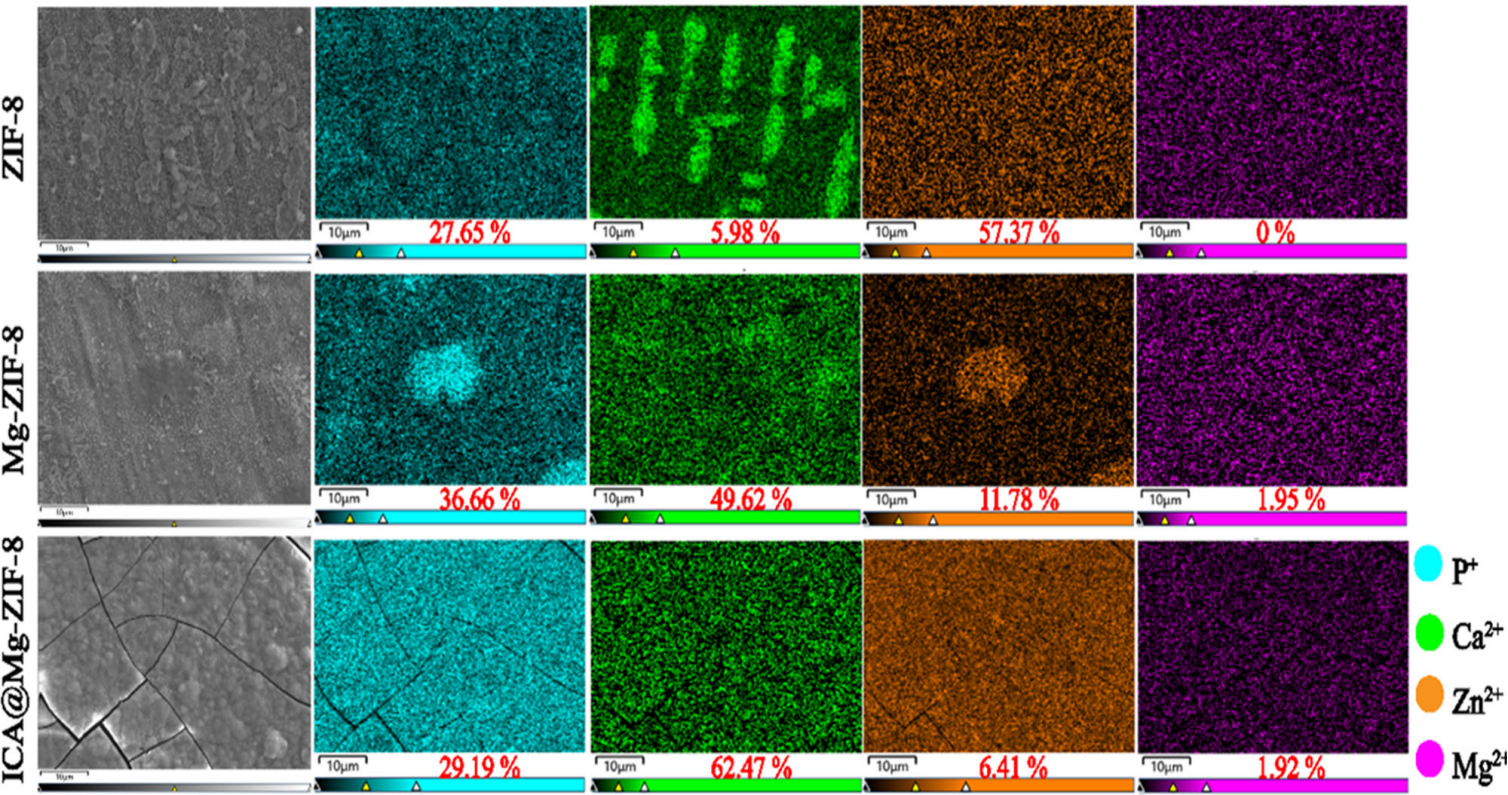
EDX-Mapping of the ZIF-8, Mg-ZIF-8 and ICA@Mg-ZIF-8 after 7 days of mineralization

After mineralization, the contents of various elements account for as shown in Fig. 4. The Ca and P contents on ICA@Mg-ZIF-8 was greater than those of ZIF-8 and Mg-ZIF-8, so the mineralization efficiency of ICA@Mg-ZIF-8 was the highest. The content of Ca and P gradually exceeded that of Zn after 7 days of mineralization, indicating that a steady flow of Ca and P ions were precipitated from SBF and deposited on the mineralized site or the formed calcium and phosphorus deposits. Therefore, ICA@Mg-ZIF-8 can induce the uniform deposition of calcium and phosphorus in the crystal structure, and with the increase of time, the formed mixed minerals appeared agglomeration and enlargement.

## Conclusion

From this work, the particle size of ZIF-8 was increased by magnesium doping and ICA loading. Mg^2+^ in this magnesium-doped ZIF-8 can promote the mineralization of bone marrow mesenchymal stem cells. The nanomaterial of magnesium doping ZIF-8 loaded with ICA performed bone promoting properties and good antibacterial activity.
